# Immune phenotypes and checkpoint molecule expression of clonally expanded lymph node-infiltrating T cells in classical Hodgkin lymphoma

**DOI:** 10.1007/s00262-022-03264-8

**Published:** 2022-08-10

**Authors:** Alexej Ballhausen, Amin Ben Hamza, Carlotta Welters, Kerstin Dietze, Lars Bullinger, Hans-Peter Rahn, Sylvia Hartmann, Martin-Leo Hansmann, Leo Hansmann

**Affiliations:** 1 Charité - Universitätsmedizin Berlin, corporate member of Freie Universität Berlin und Humboldt-Universität zu, Augustenburger Platz 1, 13353 Berlin, Germany; 2 German Cancer Consortium (DKTK), Berlin, Germany; 3 German Cancer Research Center (DKFZ), Heidelberg, Germany; 4 Preparative Flow Cytometry, Max-Delbrück-Centrum für Molekulare Medizin, Berlin, Germany; 5 Dr. Senckenberg Institute of Pathology, Goethe University Frankfurt, Frankfurt am Main, Germany; 6 Frankfurt Institute of Advanced Studies, Frankfurt am Main, Germany; 7 Institute of General Pharmacology and Toxicology, Goethe University Frankfurt, Frankfurt am Main, Germany

**Keywords:** Hodgkin lymphoma, Lymph node-infiltrating T cells, Immune checkpoint blockade, Lymphoma immunology, Single cell technologies, T cell receptor sequencing

## Abstract

Lymph node-infiltrating T cells have been of particular interest in classical Hodgkin lymphoma (cHL). High rates of complete therapeutic responses to antibody-mediated immune checkpoint blockade, even in relapsed/refractory patients, suggest the existence of a T cell-dominated, antigen-experienced, functionally inhibited and lymphoma-directed immune microenvironment. We asked whether clonally expanded T cells (1) were detectable in cHL lymph nodes, (2) showed characteristic immune phenotypes, and (3) were inhibited by immune checkpoint molecule expression. We applied high-dimensional FACS index sorting and single cell T cell receptor αβ sequencing to lymph node-infiltrating T cells from 10 treatment-naïve patients. T cells were predominantly CD4^+^ and showed memory differentiation. Expression of classical immune checkpoint molecules (CTLA-4, PD-1, TIM-3) was generally low (< 12.0% of T cells) and not different between CD4^+^ and CD8^+^ T cells. Degrees of clonal T cell expansion varied between patients (range: 1–18 expanded clones per patient) and was almost exclusively restricted to CD8^+^ T cells. Clonally expanded T cells showed non-naïve phenotypes and low checkpoint molecule expression similar to non-expanded T cells. Our data suggest that the therapeutic effects of immune checkpoint blockade require mechanisms in addition to dis-inhibition of pre-existing lymphoma-directed T cell responses. Future studies on immune checkpoint blockade-associated effects will identify molecular T cell targets, address dynamic aspects of cell compositions over time, and extend their focus beyond lymph node-infiltrating T cells.

**Supplementary Information:**

The online version contains supplementary material available at 10.1007/s00262-022-03264-8.

## Introduction

Classical Hodgkin lymphoma (cHL) is a B cell malignancy characterized by rare Hodgkin Reed-Sternberg (HRS) cells surrounded by dominating T cells, non-malignant B cells, macrophages, and innate immune cells [1]. The microenvironment is critical for disease development but also renders cHL the cancer type currently most susceptible to antibody-mediated immune checkpoint blockade. Interference with programmed death (PD-) 1 – PD-L1 interactions is highly effective even in relapsed/refractory settings and recent data suggest additional activity of antibodies against cytotoxic T lymphocyte associated protein (CTLA-) 4 and lymphocyte activating gene (LAG-) 3 in subsets of patients [2, 3]. Cell populations critically involved in immune checkpoint blockade-mediated effects remain matters of debate.

In solid malignancies, efficacy of PD-1-blockade relies on activation of CD8^+^ cytotoxic T cells in the tumor microenvironment [4]. T cells represent obvious direct targets of immune checkpoint blockade and previous studies investigated differentiation states and checkpoint molecule expression of lymph node-infiltrating and peripheral blood T cells, especially in relapsed/refractory cHL [5, 6]. However, roles of locally pre-existing lymphoma-infiltrating T cells are incompletely understood and combined information on clonal T cell expansion and high-dimensional immune phenotypes at single cell resolution are not available.

Given that clonal expansion defines functional units of T cells and is potentially driven by chronically persistent lymphoma-associated antigens, we asked at the single cell level whether cHL-infiltrating T cells show clonal expansion and immune phenotypes characteristic of functional exhaustion, which could potentially be reversed by immune checkpoint blockade.

## Materials and methods

### Patients

The study includes lymph node specimens of 10 patients with treatment-naïve cHL; median age, 24 years; 5 male, 5 female patients; 6 nodular sclerosis, 4 mixed cellularity subtype; 2 patients Ann Arbor stage II, 4 stage III, 2 stage IV; from 2 patients, staging was not available. All cases were negative for Epstein-Barr virus (EBV) infection. The research was approved by the local ethics committee, conducted in accordance with the Declaration of Helsinki, and written informed consent was obtained from all patients. All cases were reviewed by SH and MLH.

### Fluorescence-activated cell sorting

Lymph node cell suspensions were stained with multi-parameter antibody panels (Suppl. Tab. 1) according to the manufacturers’ instructions. Single cell index sorting into 96-well plates pre-filled with OneStep RT-PCR buffer (Qiagen) for T cell receptor (TCR) αβ and phenotype sequencing was done as described previously [7]. Index sorting guaranteed highly accurate 13-dimensional immune phenotyping of every single sorted cell at the protein level with accurately assigned immune phenotypes in > 99% of sorted T cells [7]. All cells were sorted using a FACSAria™ Fusion high-speed cell sorter (BD Biosciences) equipped with a 70 µm nozzle and were frozen at -80 ºC immediately after sorting until further processing. Multi-parameter immune phenotyping was performed on all ten patients in the study; sufficient material for single cell index sorting was available from nine out of ten patients. Index sorting data were exported from FACSDiva software (BD Biosciences) as “comma-separated values” (.csv) files according to the manufacturer’s instructions and combined with single cell sequencing data.

### T cell receptor αβ, cytokine, and transcription factor sequencing

Sorted cells were thawed; single cell nucleic acid amplification, library preparation, molecular barcoding, MiSeq (Illumina) TCRαβ and phenotype sequencing were done as previously described [8, 9]. In short, reverse transcription PCR and first amplification were performed with gene-specific primers followed by two nested PCR amplifications, which introduced DNA barcodes specific for plate and well of origin of each single transcript. PCR products were purified by gel electrophoresis and sequenced on an Illumina MiSeq instrument (Illumina) using MiSeq Reagent Kits v2, 500 cycles (Illumina) for paired-end sequencing. Computational scripts for sequencing data processing are publicly available at “https://github.com/HansmannLab/TRECA”. Cytokines and transcription factors were considered expressed in single cells if we detected more than 10 reads per cell for the respective cytokine or transcription factor [8]. T cell clones were defined expanded if we detected at least two individual cells with identical TCRαβ CDR3 amino acid sequences within the same lymph node.

## Results

### Immune phenotypes of classical Hodgkin lymphoma-infiltrating T cells

**Fig. 1:**
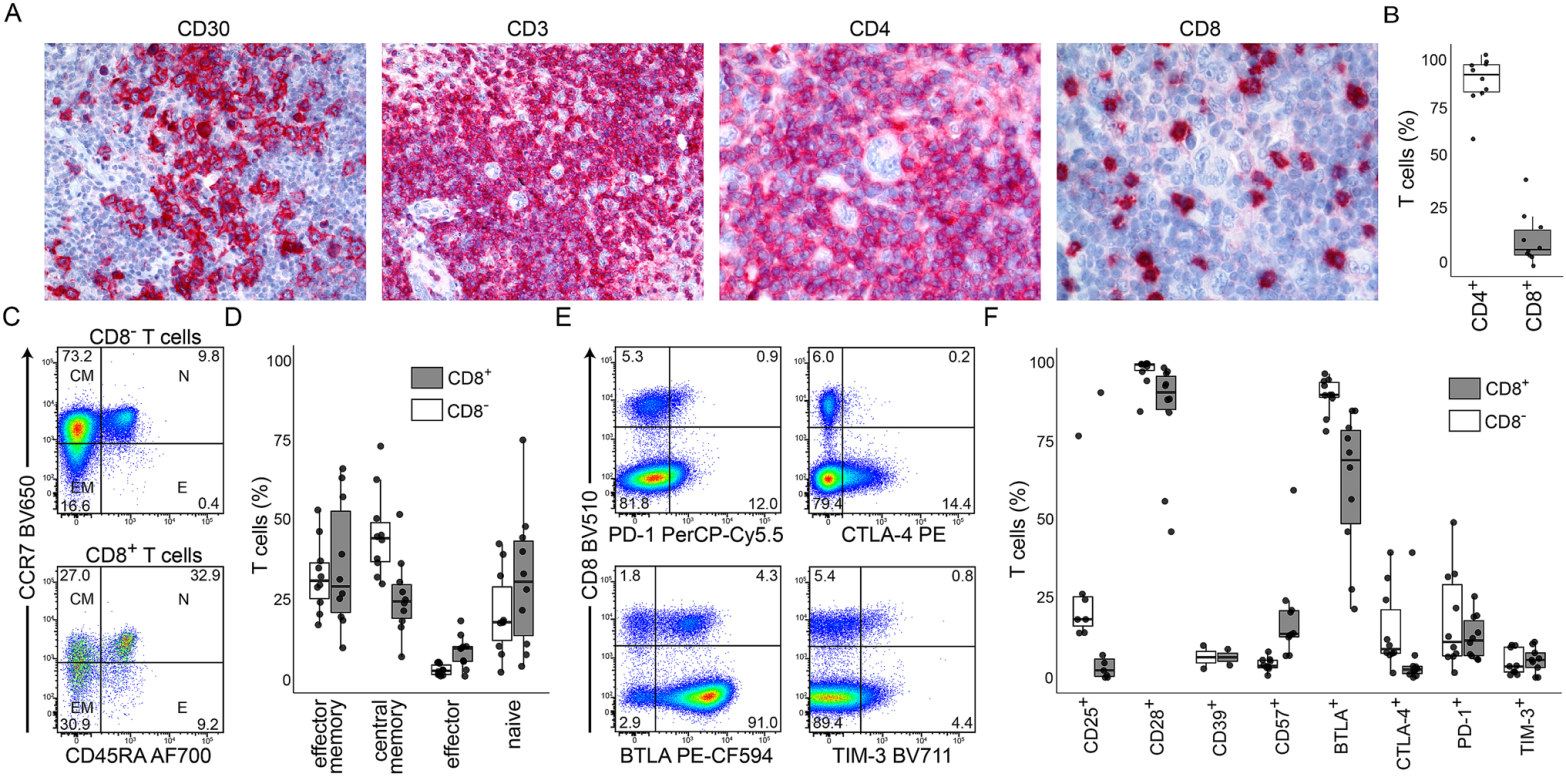
Immune phenotypes of lymph node-infiltrating T cells. **A** Immuno histology of one representative classical Hodgkin lymphoma lymph node. Data from patient CHL003 (mixed cellularity subtype) are shown as a representative example. **B** Frequencies of CD4^+^ and CD8^+^ cells among total αβ T cells within Hodgkin lymphoma lymph nodes determined by flow cytometry. Data points indicate n=10 individual patients. **C** Identification of naïve “N”, effector “E”, central memory “CM”, and effector memory “EM” populations within CD8^+^ and CD8^-^ T cells from patient CHL003 as an example. Plots are pre-gated on live single TCRαβ^+^ cells (gating strategy in Suppl. Fig. 1). **D** Frequencies of T cell subpopulations as shown in (**C**) within all patients. Data points indicate n=10 individual patients. **E** Detection of checkpoint molecule expression on αβ T cells from patient CHL003 as an example. Plots are pre-gated on live single TCRαβ^+^ cells. Gates for PD-1, CTLA-4, and TIM-3 were defined based on expression on non-T cells. **F** Checkpoint molecule/functional marker expression on lymph node-infiltrating αβ T cells determined by flow cytometry as shown in (**E**). Data points indicate individual patients. Data are representative for n=10 individual patients, except for CD39 (n=2), CD25 (n=7), and TIM-3 (n=9). In all box plots, lower and upper hinges correspond to the 25th and 75th percentiles, whiskers extend from the hinges to largest and smallest values but no further than 1.5 x interquartile range, all other data points are shown as outliers. AF indicates AlexaFluor; BV, Brilliant Violet; PerCP, peridinin chlorophyll protein; Cy, cyanine; PE, phycoerythrin; and CF, cyanine-based fluorescence.

Lymph nodes were dominated by infiltrating T cells (Fig. 1a), the majority of which were CD8^−^ (Fig. 1a and b). As expected, CD8^−^ T cells showed dominant central memory (CD45RA^−^CCR7^+^) and effector memory (CD45RA^−^CCR7^−^) differentiation while CD8^+^ T cells were almost evenly distributed between naïve (CD45RA^+^CCR7^+^), central memory, and effector memory compartments (Fig. 1c and d). Frequencies of effector cells (CD45RA^+^CCR7^−^) were in median below 10% of CD8^+^ and CD8^−^ T cells (Fig. 1d).

Given the high therapeutic response rates to immune checkpoint blockade, we focused on checkpoint molecule expression on lymph node-infiltrating T cells. CTLA-4, PD-1, and TIM-3 were expressed in median on no more than 12% of T cells; however, we observed a trend towards higher expression on CD8^−^ T cells with substantial variation and PD-1 expression on up to 49% of CD8^−^ T cells in selected patients (Fig. 1e and f). CTLA-4 expression was more frequently detectable on CD8^−^ T cells, a trend also observed for CD25 expression (Fig. 1e and f), which indicated regulatory T cell differentiation, commonly observed in the cHL microenvironment [10]. In contrast to PD-1, CTLA-4, and TIM-3, BTLA was expressed on the majority of CD8^+^ and CD8^−^ T cells (Fig. 1e and f). Apart from classical immune checkpoint molecules, CD57 was expressed on less than 25% of T cells and CD28 expression could be detected on more than 90% of T cells, which is consistent with non-effector differentiation in the majority of T cells (Fig. 1d and f). CD39 expression, which can indicate neo-antigen-specific T cells [11], was generally low (Fig. 1f).

In summary, lymph node-infiltrating T cells showed dominant non-effector differentiation. Expression of the immune checkpoint molecules PD-1, CTLA-4, and TIM-3, or CD57, which had been associated with functional exhaustion, was rare in the majority of patients.

### Clonally expanded T cells rarely express immune checkpoint molecules

Clonal expansion is an antigen-driven process that defines functional T cell units. We hypothesized that chronically persistent cHL-associated antigens could drive clonal expansion and functional exhaustion of disease-specific lymph node-infiltrating T cells.

**Fig. 2:**
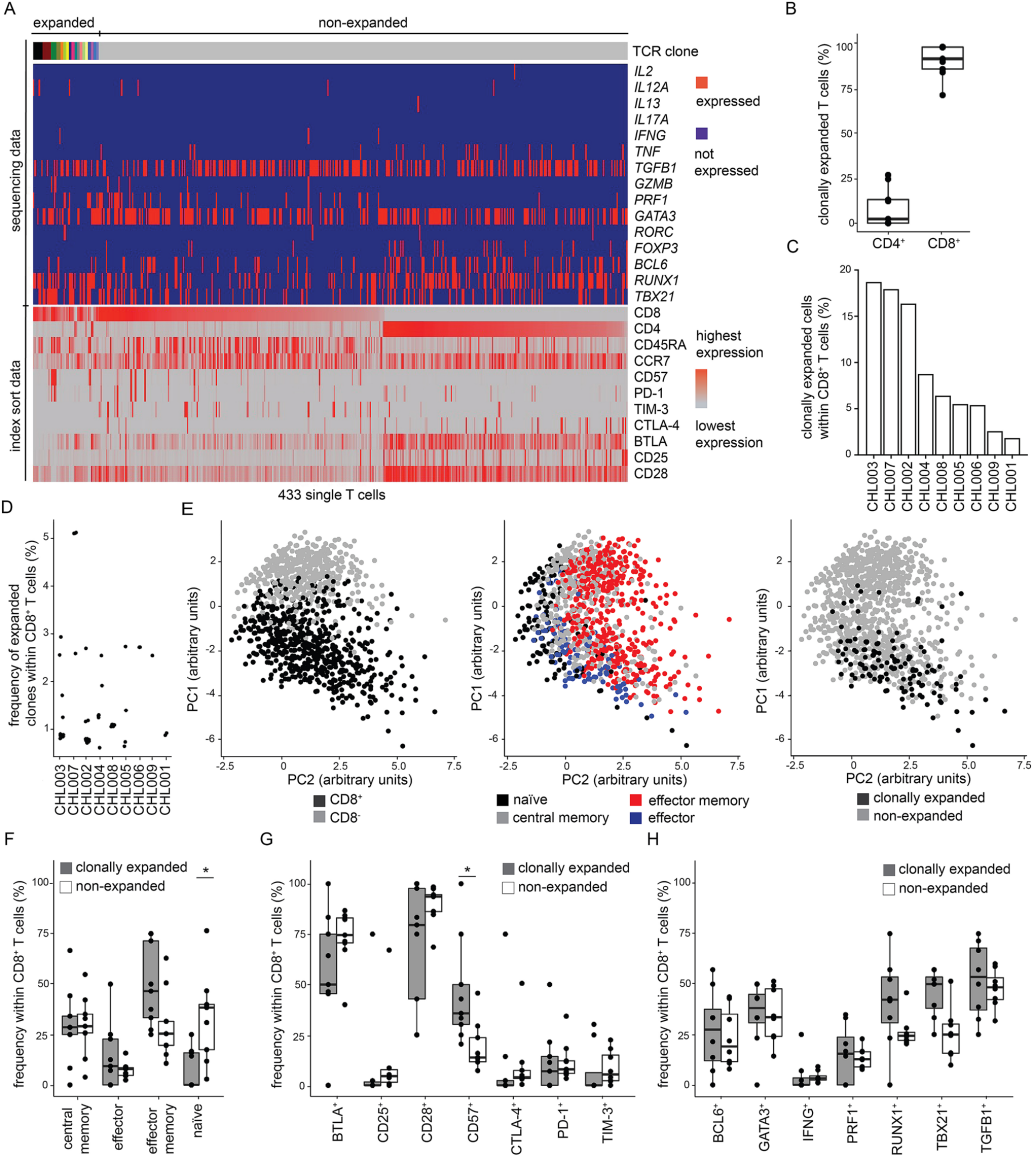
Clonal T cell expansion-associated immune phenotypes. **A** Sequencing of TCRαβ, transcription factor, and cytokine genes from amplified cDNA of single FACS-sorted T cells. The heatmap shows data of patient CHL003 as an example (heatmaps of all patients in Suppl. Fig. 2). Single cell data are arranged in columns with each column representing one single cell. The top bar indicates TCR sequences; adjacent columns with the same color in the top bar indicate single cells with identical CDR3 amino acid sequences of TCRαβ genes. T cell clones were defined expanded if we detected at least two cells with identical TCRαβ CDR3 amino acid sequences within individual lymph nodes. The lower part of the heatmap visualizes corresponding FACS index sort data (Suppl. Fig. 1 for sort gates). **B** Expression of CD4 and CD8 on clonally expanded lymph node-infiltrating T cells. Data points indicate individual patients. **C** Frequencies of clonally expanded T cells within total CD8^+^ T cells. **D** Sizes of expanded clones measured by frequencies within CD8^+^ T cells. Data points indicate individual expanded T cell clones. **E** Principal component analysis taking into account differentiation state and individual marker expression (detailed list in Suppl. Tab. 3) of all sequenced T cells. Data points indicate individual cells. **F** Differentiation of clonally expanded and non-expanded CD8^+^ T cells. **G** Surface marker expression on clonally expanded and non-expanded CD8^+^ T cells determined by FACS index sorting. Individual data points indicate n=9 patients, except for CD25 (n=6). **H** Parameters determined by single cell gene expression within clonally expanded and non-expanded CD8^+^ T cells. Visualized markers reached expression in at least 5% of T cells (Suppl. Fig. 3 for all markers). Individual data points indicate n=8 patients. PC indicates principal component. In all box plots, data points indicate individual patients; lower and upper hinges correspond to the 25th and 75th percentiles, whiskers extend from the hinges to largest and smallest values but no further than 1.5 x interquartile range, all other data points are shown as outliers. Statistical significance was calculated using the Wilcoxon Rank-Sum Test and corrected for multiple testing by Bonferroni adjustment, *p<0.05

From nine out of ten patients, we determined clonal T cell expansion-associated immune phenotypes, transcription factor and cytokine expression within 3 317 single lymph node-infiltrating T cells (median: 369 T cells per patient) (Fig. 2). To account for the physiologic predominance of CD4^+^ T cells in cHL lymph nodes, gates for single cell index sorting were adjusted to select approx. equal numbers of CD4^+^ and CD8^+^ T cells of each patient for sequencing (Suppl. Fig. 1). Sequencing and flow cytometry phenotyping data of patient CHL003 were illustrated as an example (Fig. 2a; Suppl. Fig. 2 for all patients). Absolute numbers of expanded clones were low (median: 4 clones per patient, range: 1–18 clones per patient, Suppl. Tab. 2). Clonal T cell expansion was almost exclusively (89% of expanded clones) restricted to CD8^+^ compartments (Fig. 2b); therefore, we focused further analyses on CD8^+^ T cells. Frequencies of clonally expanded T cells varied between patients (Fig. 2c), and dominant clones accounted for 1–5% of CD8^+^ T cells (Fig. 2d).

Principal component analysis of T cell immune phenotypes (Fig. 2e) resulted in formation of two major clusters associated with CD8 expression and partly overlapping naïve, memory, central memory, and effector memory compartments (identified by the same criteria as in Fig. 1c) (Fig. 2e left and middle panel). As expected, clonally expanded T cells mapped to CD8^+^ clusters (fig. 2e right panel). Naïve phenotypes were significantly enriched in non-expanded T cells while we observed a trend towards effector and effector memory differentiation within clonally expanded T cells (Fig. 2f). Accordingly, CD57 was more frequently expressed on clonally expanded T cells. Expression of immune checkpoint molecules, cytokines, or transcription factors was not significantly different between clonally expanded and non-expanded T cells (Fig. 2g and h, Suppl. Fig. 3 for all parameters).

To investigate whether HRS cells could be potential direct targets of clonally expanded CD8^+^ T cells, we determined HLA class-I expression within four patients of whom remaining lymph node specimens were available. HLA class-I expression was detectable on HRS cells of all four patients but was lower than on B or T cells from the same lymph nodes (Suppl. Fig. 4).

In conclusion, clonally expanded T cells in cHL lymphoma lymph nodes are rare, almost exclusively CD8^+^, show non-naïve immune phenotypes, and only in the minority express classical immune checkpoint molecules.

## Discussion

Classical Hodgkin lymphoma is a prime example of therapeutically successful immune checkpoint blockade. PD-L1 expression has been shown to be genetically imprinted in HRS cells and antibodies interfering with PD-1/PD-L1 interactions have been of particular therapeutic efficacy [12, 13]. Considering clonal T cell expansion and immune checkpoint molecule expression to be driven by (chronic) antigen stimulation, we determined immune phenotypes of clonally expanded T cell compartments in treatment-naïve Hodgkin lymphoma lymph nodes.

Dominant non-effector differentiation of lymph node-infiltrating T cells in our study was representative of data from larger cohorts [14, 15]. Clonal T cell expansion was almost exclusively restricted to CD8^+^ compartments, which was surprising since PD-1 and CTLA-4 tended to be more frequently expressed on CD8^−^ T cells and therapeutic effects of PD-1 blockade had previously at least in parts been attributed to CD4^+^ T cells [16]. Notably, in diffuse large B cell lymphoma, clonal T cell expansion has also been shown to predominantly occur in the CD8^+^ compartment and dominant clonotypes were shared between lymphoma tissue and peripheral blood [17]. Whether clonally expanded CD8^+^ T cells in our cohort recognized antigens presented on HRS cells cannot be concluded from our data. Frequent absence or low expression of HLA class-I, especially in EBV-negative cases [18, 19], makes HRS cells unlikely direct targets of CD8^+^ T cell-mediated cytotoxicity. All cases of our cohort were EBV-negative and HLA class-I expression was reduced when compared to B cells but reliably detectable on HRS cells. Physiological consequences of reduced HLA class-I expression on antigen presentation and potential lymphoma-specific T cell responses have to be determined in future studies.

Besides Hodgkin lymphoma and other malignancies, clonal T cell expansion has also been observed in lymph nodes and peripheral blood of healthy individuals [20] and cannot be considered a proof of lymphoma-specificity. Accurate definition of lymphoma-reactive T cell clones requires further development of currently emerging technologies for high-throughput identification of unknown TCR target epitopes [21–23].

We demonstrate that clonally expanded T cells in cHL lymph nodes are rarely inhibited by PD-1 expression questioning our current understanding of mechanisms underlying clinically efficient immune checkpoint blockade. Our data suggest that therapeutic responses to immune checkpoint blockade do not rely on an a priori existence of lymph node-resident lymphoma-specific T cells, which is in line with findings from solid malignancies [24]. Our findings are supported by studies that demonstrate absence of cytotoxic T cell activation after immune checkpoint blockade [25]. Furthermore, high motility and frequent cell–cell interactions of PD-1^+^ T cells suggest that immune cell compartments in cHL are highly dynamic [26] and likely to change substantially upon checkpoint blockade-mediated perturbation. Future research on critical mechanisms of immune checkpoint blockade in cHL will (1) identify targets of lymph node-infiltrating T cells, (2) study motility and spatial distribution of selected (immune) cell populations, and iii) identify and track cells attracted into cHL lymph nodes as a consequence of therapy.

## Supplementary Information

Below is the link to the electronic supplementary material.

Supplementary file1 (PDF 11746 KB)

## Data Availability

Detailed flow cytometry and sequencing data can be found in a data supplement. Raw data will be made available upon reasonable request.
