# Supplementary material for: Immune phenotypes and checkpoint molecule expression of clonally expanded lymph node-infiltrating T cells in classical Hodgkin lymphoma
Source: Cancer Immunol Immunother. 2022 Aug 10;72(2):515–21. doi: 10.1007/s00262-022-03264-8 (PMC9870823; doi:10.1007/s00262-022-03264-8)
Supplement: Supplementary file 1 — Supplementary file1 (PDF 11746 KB) [file 262_2022_3264_MOESM1_ESM.pdf]

Supplementary Information

Supplementary Table 1

Target	*Clone*	*Fluorescent dye*	*Vendor*	*Application*
CD8	RPA-T8	BV510	BioLegend	I
CD57	HCD574	FITC	BioLegend	I
PD-1	EH12.2H7	PerCP-Cy5.5	BioLegend	I
BTLA	J168-570	PE-CF594	BD Biosciences	I
TCRαβ	IP26	APC	BioLegend	I/H
CD45RA	HI100	AF700	BioLegend	I
CD38	HIT2	PE-Cy7	BioLegend	I
CD39	A1	PE-Cy7	BioLegend	I
CD25	CD25-4E3	PE-Cy7	eBioscience	I
CTLA-4	BNI3	PE	Invitrogen	I
CCR7	G043H7	BV650	BioLegend	I
CD28	CD28.2	BV421	BioLegend	I
TIM-3	7D3	BV711	BD Biosciences	I
CD4	SK3	APC-Fire750	BioLegend	I
Live/dead		Zombie Yellow	BioLegend	I
CD19	HIB19	BV650	BioLegend	H
CD30	Ki-2	FITC	Miltenyi	H
HLA class-I	W6/32	PE	BioLegend	H
Isotype control	20102	PE	R&D Systems	H

Supplementary Table 1 Flow cytometry reagents

All reagents were used according to the manufacturers' instructions and titrated before application in 13-parameter panels. For CHL002 and CHL004, we used CD39 instead of CD25 antibody. For CHL008, CD38 was used instead of CD25, and no TIM-3 staining was done in this sample. APC indicates allophycocyanin; BV, Brilliant Violet; FITC, fluorescein isothiocyanate; PerCP, peridinin chlorophyll; Cy, cyanine; PE, phycoerythrin; AF, Alexa Fluor; I, index sorting; and H, determination of HLA class-I expression.

Supplementary Table 2

Patient	*Total number of sequenced T cells*	*Non clonally expanded T cells*	*Clonally expanded T cells*	*Expanded T cell clones*
CHL001	422	418	4	2
CHL002	424	381	43	18
CHL003	433	385	48	18
CHL004	375	360	15	6
CHL005	276	265	11	4
CHL006	277	273	4	2
CHL007	341	333	8	4
CHL008	384	370	14	7
CHL009	385	382	3	1

Supplementary Table 2 Detailed clonal expansion characteristics of all sequenced T cells per patient

Supplementary Table 3

Parameter
Clonal expansion status
CD8
CD4
CD45RA
CCR7
CD57
PD-1
CTLA-4
BTLA
CD25
CD28
IFNG
TNF
TGFB1
GZMB
Central memory status
Effector status
Effector memory status
Naïve status

Supplementary Table 3 Parameters and differentiation states included for principal component analysis

Supplementary Figure 1

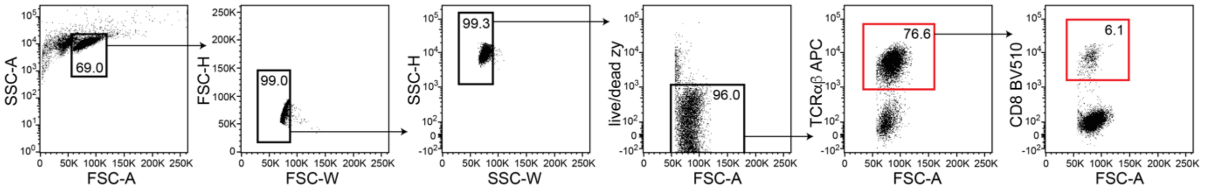

Supplementary Figure 1 Gating strategy for phenotyping and single cell index-sorting

Sequential gating on lymphocytes, single cells, live cells, αβ T cells, $CD8^+$ cells. Gates from which cells were finally sorted are indicated in red. Primarily, the TCRαβ gate was used for sorting. If frequencies of $CD8^+$ T cells were low, we sorted additional single $TCR\alpha\beta^+CD8^+$ cells to ensure representation of approx. 50% $CD8^+$ T cells among all sorted cells. The figure shows sort gates of patient CHL003 as a representative example. zy indicates zombie yellow; APC, allophycocyanin; and BV, Brilliant Violet.

Supplementary Figure 2

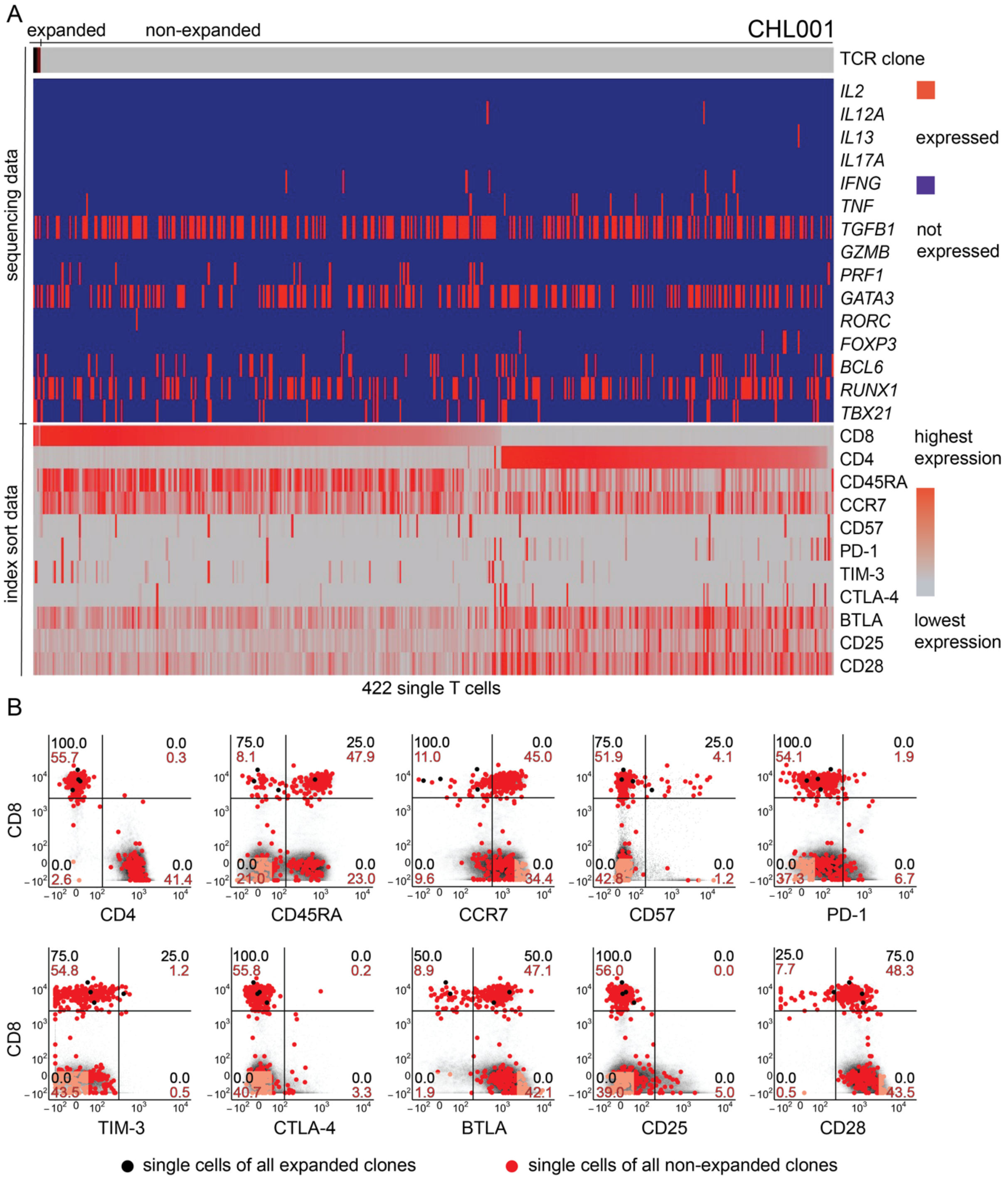

Supplementary Figure 2 continued

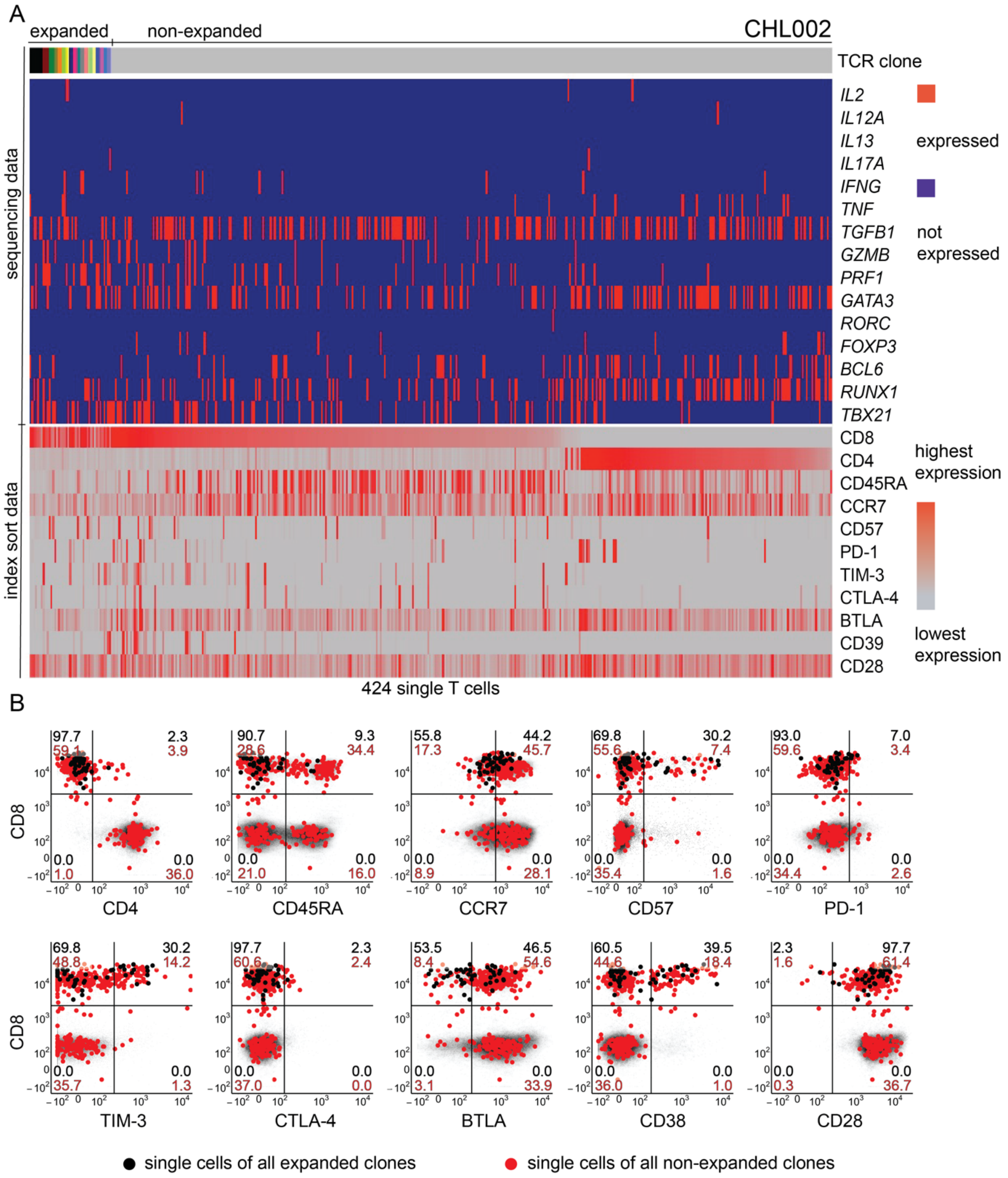

Supplementary Figure 2 continued

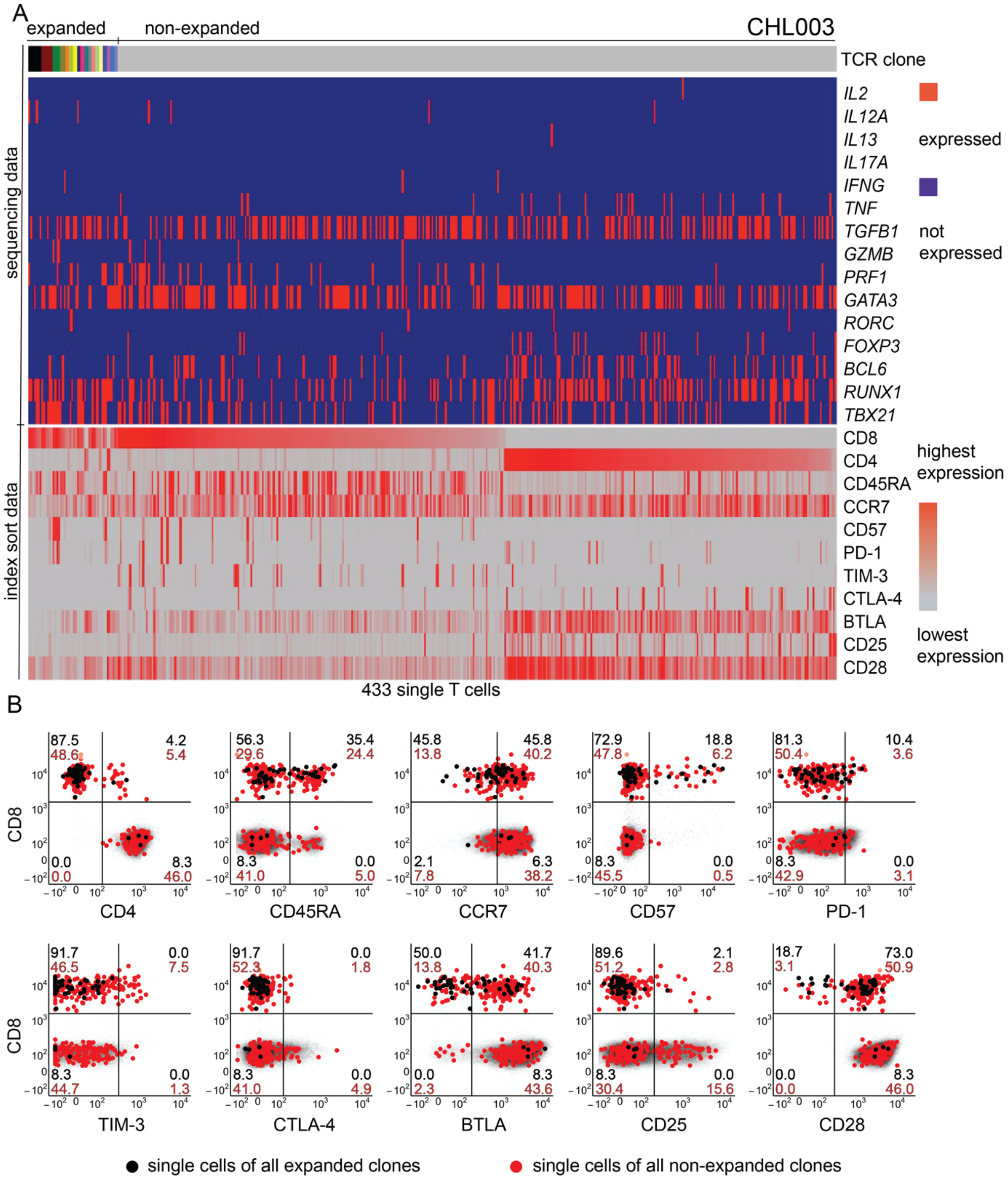

Supplementary Figure 2 continued

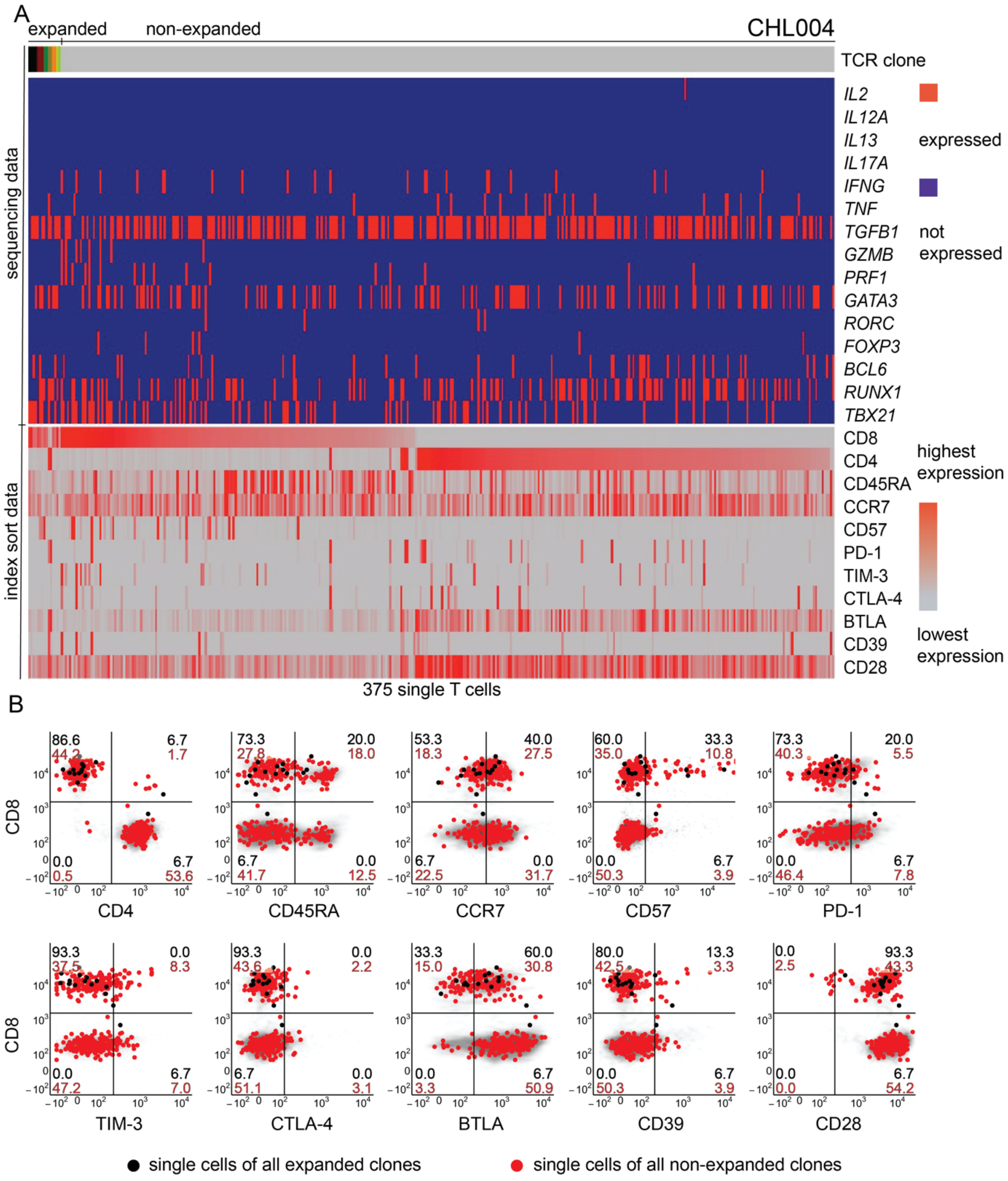

Supplementary Figure 2 continued

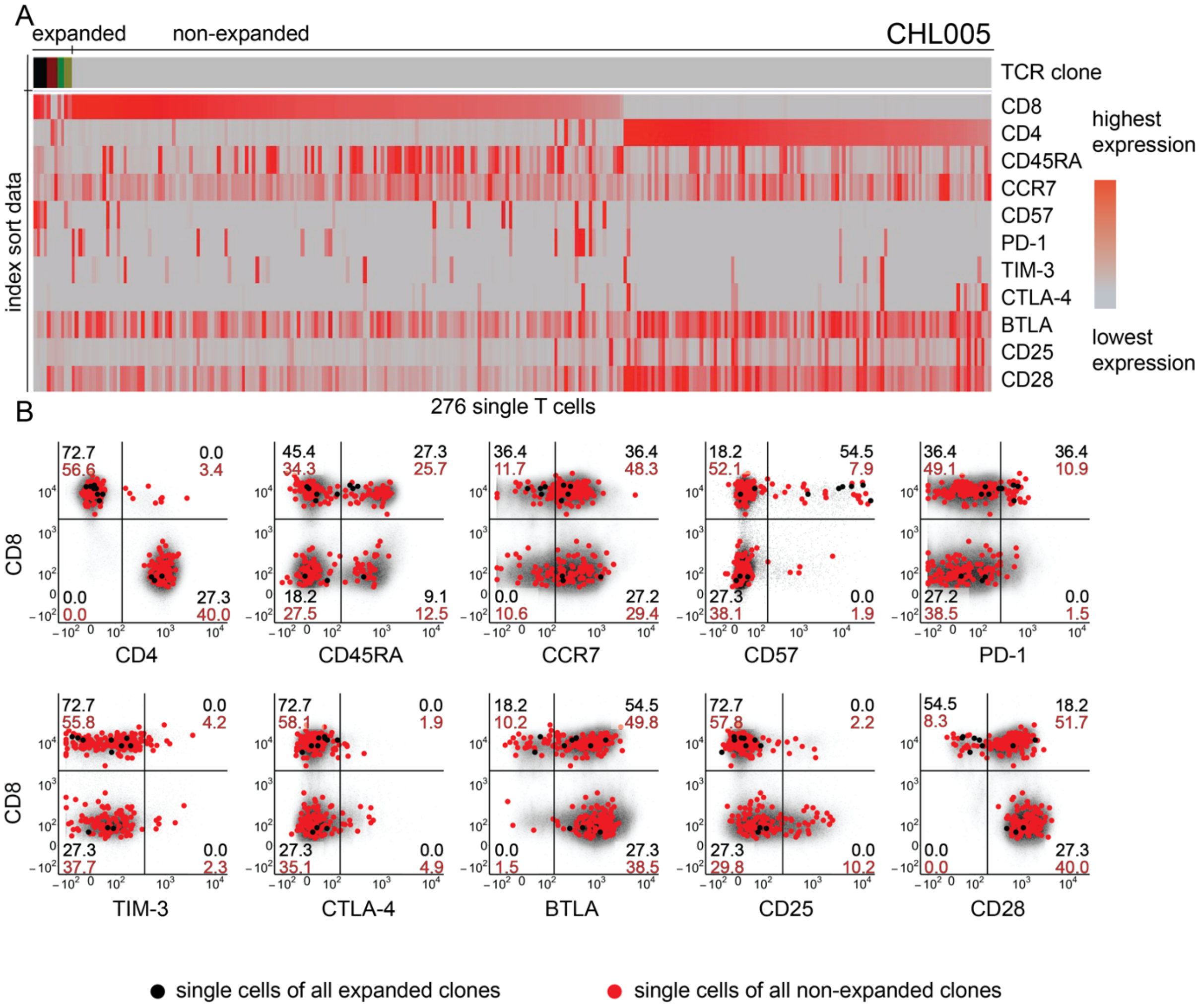

Supplementary Figure 2 continued

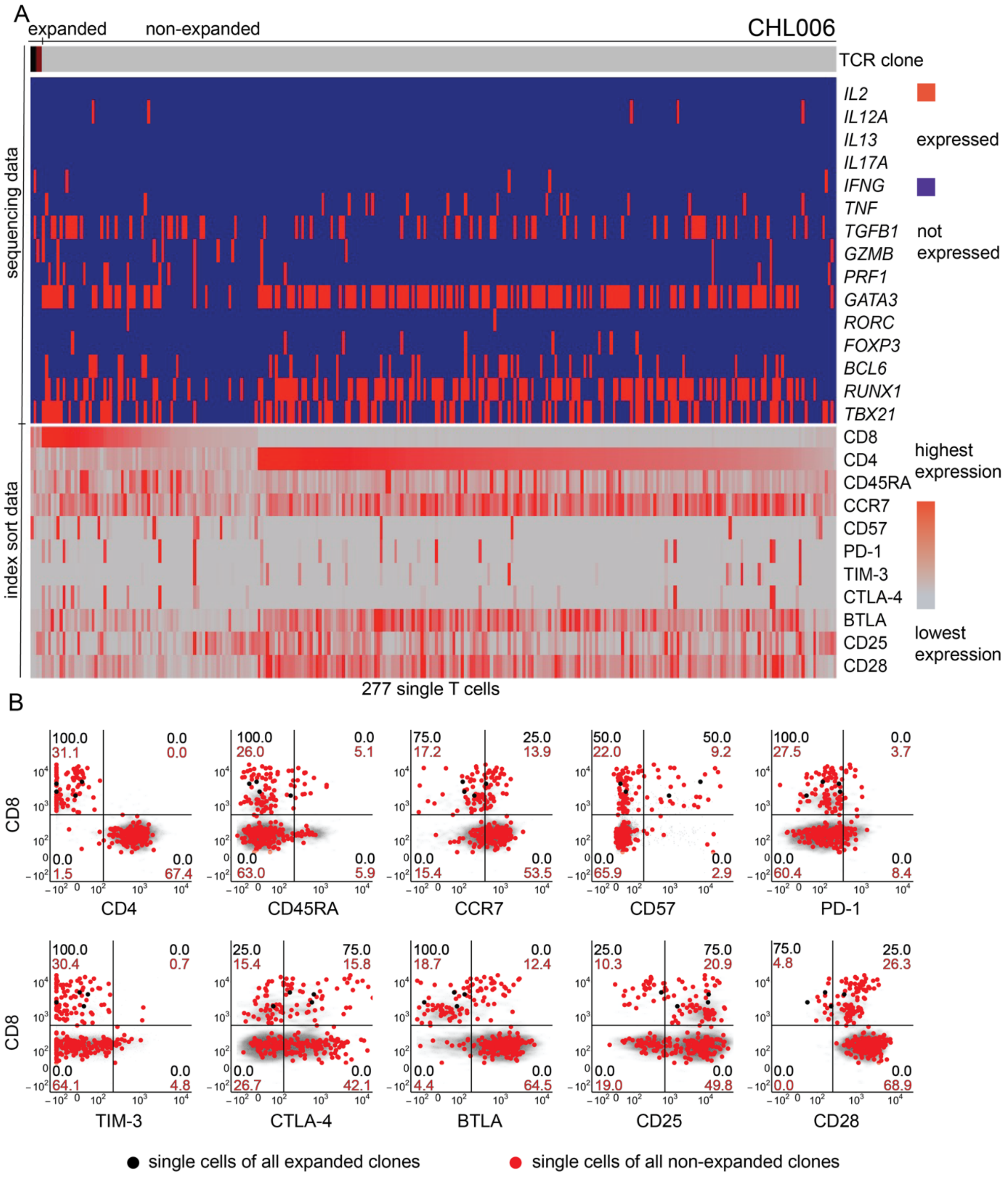

Supplementary Figure 2 continued

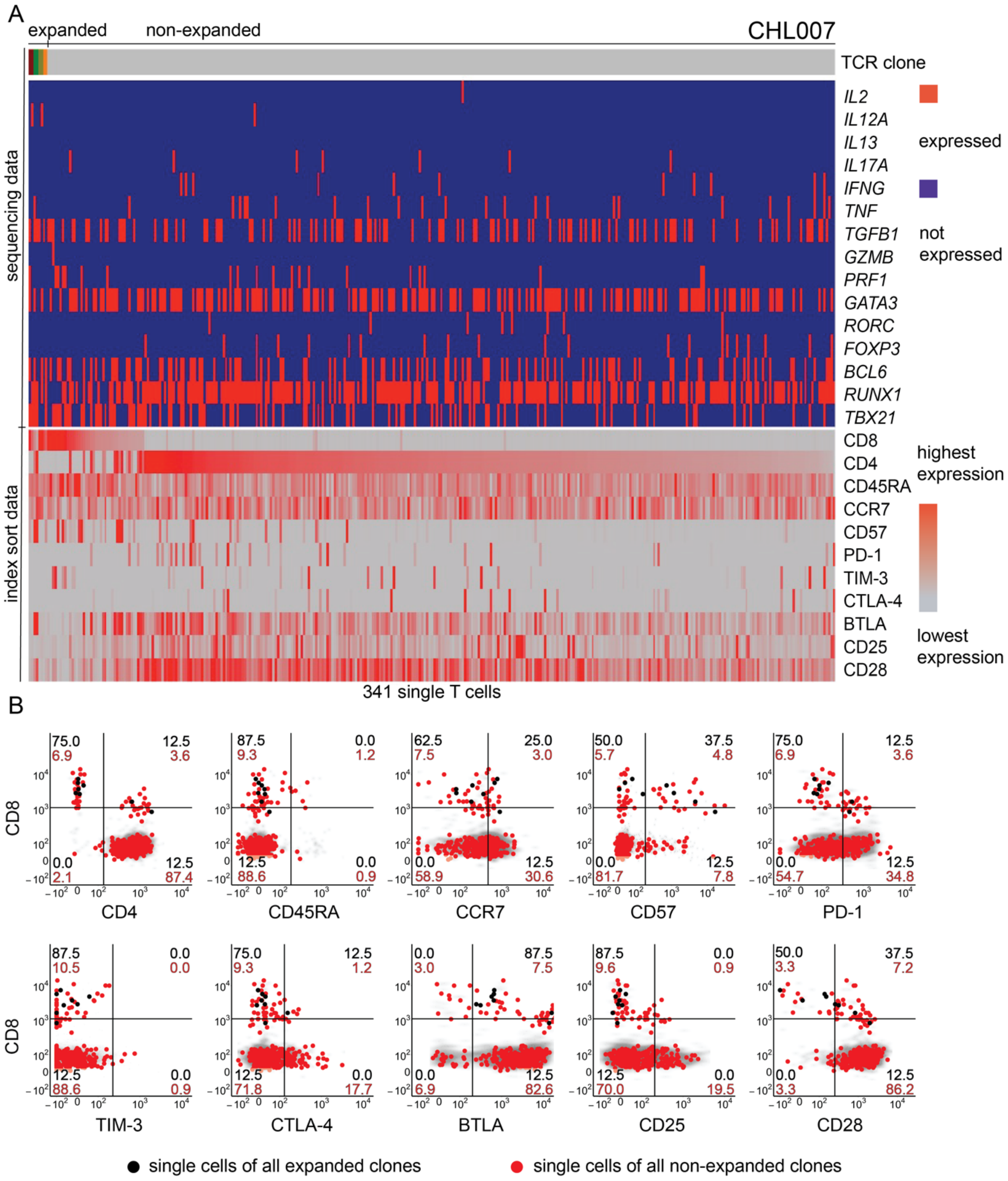

Supplementary Figure 2 continued

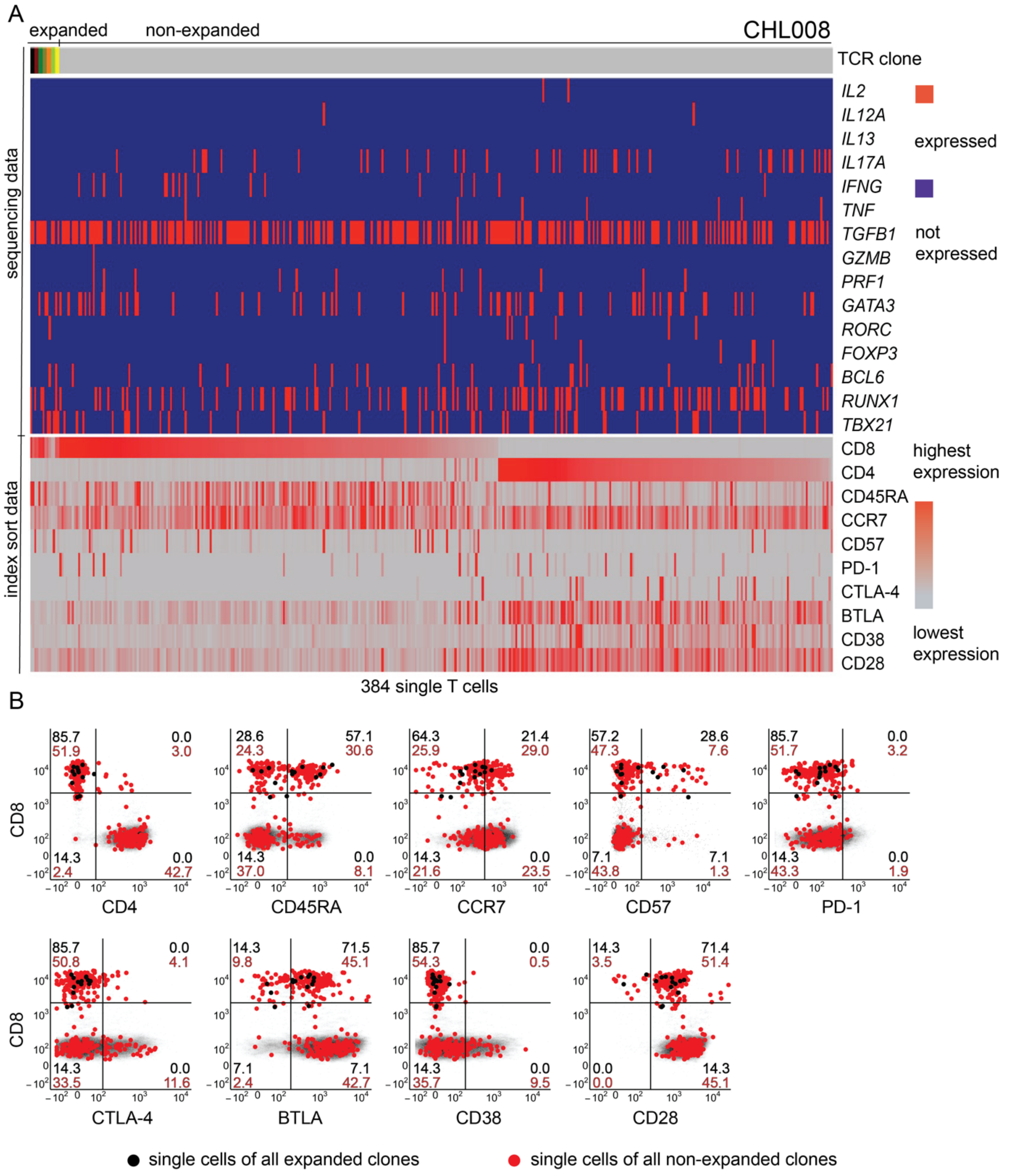

Supplementary Figure 2 continued

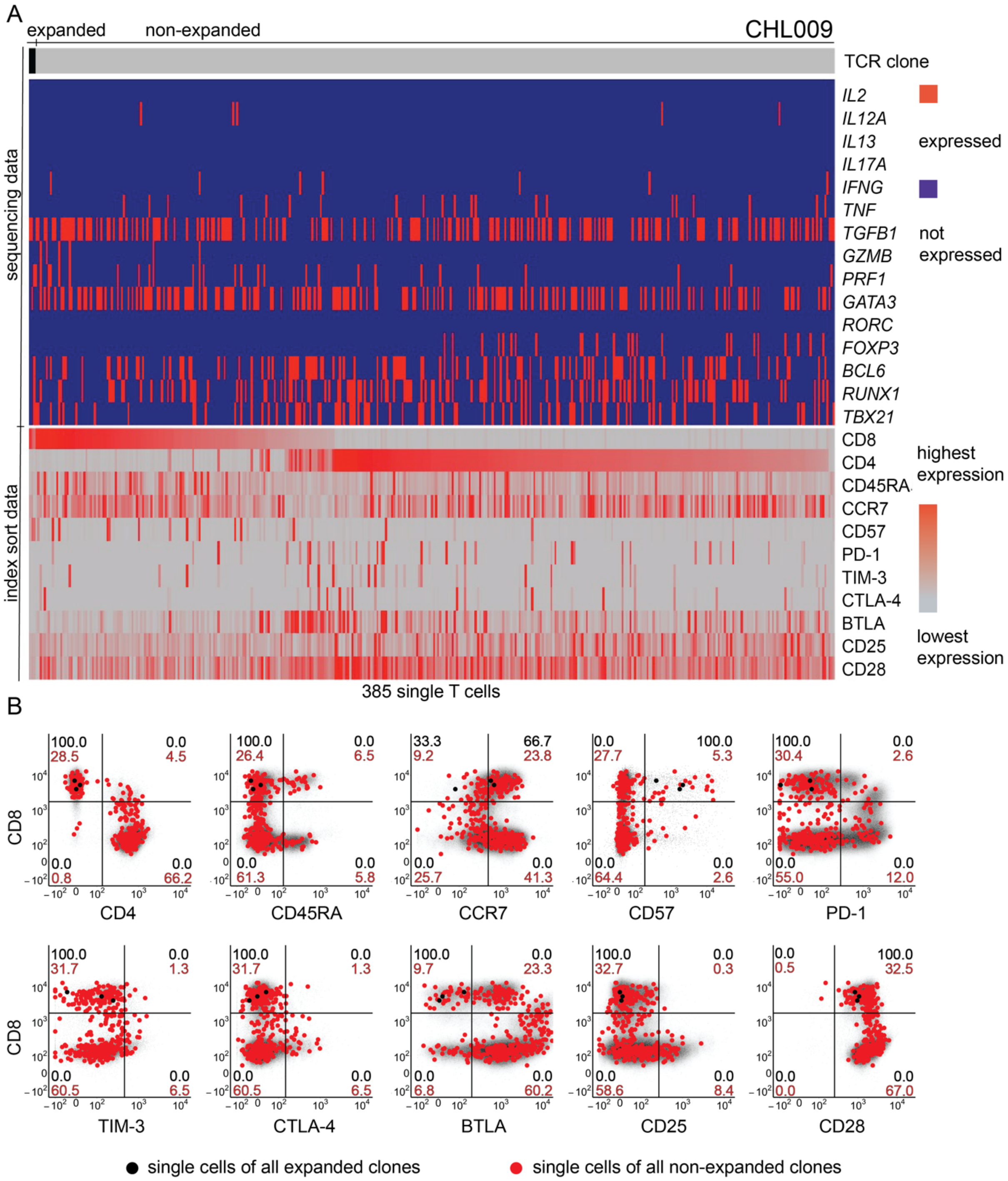

Supplementary Figure 2 Clonal expansion-associated immune phenotypes of lymph node-infiltrating T cells

(A) Sequencing of TCRαβ, transcription factor, and cytokine genes from amplified cDNA of single FACS-sorted T cells. Single cell data are arranged in columns with each column representing one single cell. The top bar indicates TCR sequences; adjacent columns with the

same color in the top bar indicate single cells with identical CDR3 amino acid sequences of TCRαβ genes. The lower part of the heatmap visualizes corresponding FACS index sort data. (B) FACS plots show immune phenotypes of expanded and non-expanded T cell clones determined by FACS index sorting. Each data point represents one single T cell belonging to an expanded (black) or non-expanded (red) clone. Data of sequenced cells (black and red) are overlaid over FACS data of total bone marrow cells of the same patients (visualized in grey) as a reference. Numbers within gates indicate percentages.

From T cells of patient CHL005, only TCRs were sequenced and immune phenotypes were determined by index sorting.

Supplementary Figure 3

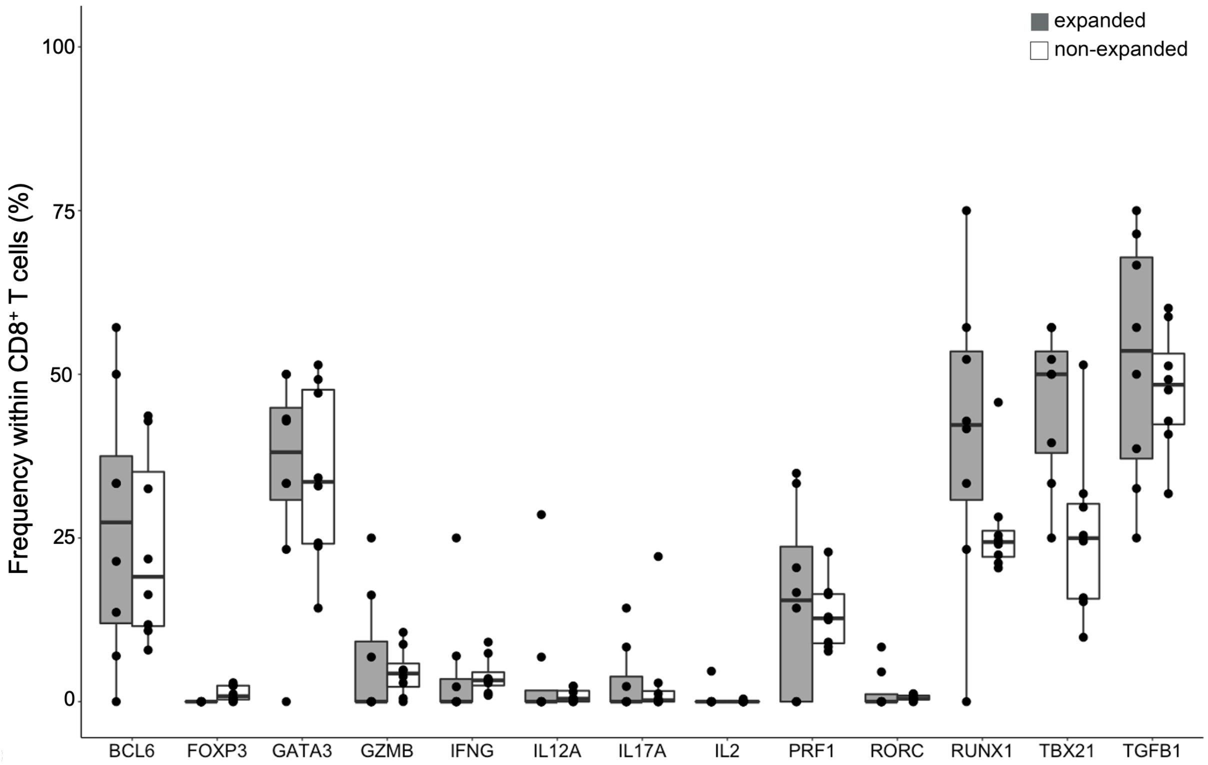

Supplementary Figure 3 Cytokine and transcription factor expression in expanded and non-expanded lymph node-infiltrating T cell clones

Single cell gene expression of cytokines and transcription factors in expanded or non-expanded $CD8^+$ T cells. Data points represent individual patients. Boxes range from 25th to 75th percentiles, lines within boxes indicate medians. Differences in marker expression between expanded and non-expanded T cell clones were not statistically significant ($p>0.05$). Statistical significance was calculated using the Wilcoxon Rank-Sum Test and corrected for multiple testing by Bonferroni adjustment.

Supplementary Figure 4

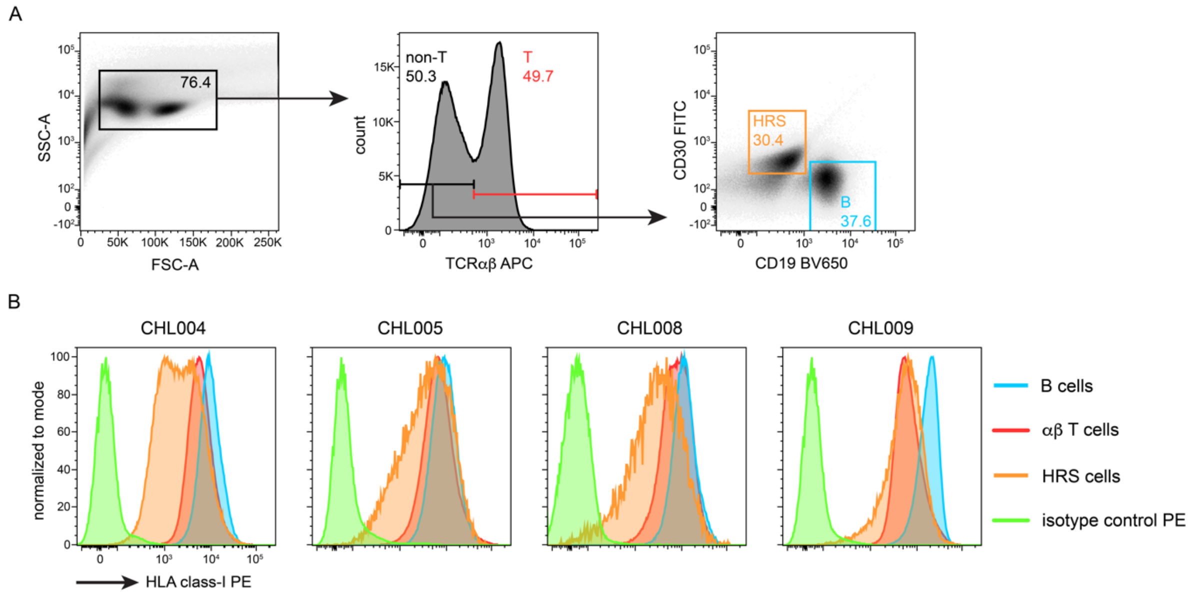

Supplementary Figure 4 HLA class-I expression on Hodgkin Reed-Sternberg cells

HLA class-I expression in lymph node cell suspensions of four patients was determined by flow cytometry. (A) shows sequential gating on nucleated cells, T/non-T cells, B cells, and HRS cells. The plots show data of patient CHL004 as an example. (B) HLA class-I expression on cell populations identified by the gating strategy illustrated in figure part A. Each plot shows data from one patient as indicated above the plot.

Numbers within gates indicate percentages; non-T, non-T cells; B, B cells; and HRS, Hodgkin Reed-Sternberg cells.
